# MULTIPLE FACTORS INFLUENCING SUCCESSFUL AGING

**DOI:** 10.1093/geroni/igz038.2303

**Published:** 2019-11-08

**Authors:** Heather Fuller

**Affiliations:** North Dakota State University, Fargo, North Dakota, United States

## Abstract

Empirically-based theories on successful aging have emphasized the multidimensional nature of aging well, including physical health and functioning, cognitive and emotional well-being, and social connectedness and engagement (e.g., Rowe & Kahn, 1987, Depp & Jeste, 2006). Yet, the field is still continuing to discover, deeply characterize, and better understand the biopsychosocial mechanisms through which varying social, physical, or cognitive activities may influence unique domains of successful aging. The current symposium builds on this growing body of research by addressing factors supporting successful aging across multiple dimensions of well-being and among a diversity of samples ranging from urban to rural, West Coast to Midwest, and community populations to professional athletes. Webster and Antonucci examine the links between social engagement and successful aging among affordable senior housing residents. They found that more frequent participation in social activities was associated with increases in life satisfaction over time. Toyama and Fuller examine how social engagement and health affect aging well, finding that older adults’ subjective health plays a more important role than objective health in maintaining social integration over time. Similarly, Turner describes the role of social networks and religiosity for health outcomes among aging NFL athletes. Finally, Casaletto and colleagues examined mechanisms underlying cognitive wellness as an aspect of successful aging. They found that engagement in both physical and cognitive activities independently support brain health and cognitive reserve in late-life. Taken together, these presentations provide a diverse and broad perspective on how varying factors influence the multiple dimensions of successful aging.

